# Combination of Proton Therapy and Radionuclide Therapy in Mice: Preclinical Pilot Study at the Paul Scherrer Institute

**DOI:** 10.3390/pharmaceutics11090450

**Published:** 2019-09-02

**Authors:** Cristina Müller, Maria De Prado Leal, Marco D. Dominietto, Christoph A. Umbricht, Sairos Safai, Rosalind L. Perrin, Martina Egloff, Peter Bernhardt, Nicholas P. van der Meulen, Damien C. Weber, Roger Schibli, Antony J. Lomax

**Affiliations:** 1 Center for Radiopharmaceutical Sciences ETH-PSI-USZ, Paul Scherrer Institute, 5232 Villigen-PSI, Switzerland; 2 Department of Chemistry and Applied Biosciences, ETH Zurich, 8092 Zurich, Switzerland; 3 Center for Proton Therapy, Paul Scherrer Institute, 5232 Villigen-PSI, Switzerland; 4 Department of Radiation Physics, Sahlgrenska Academy, University of Gothenburg, 41345 Gothenburg, Sweden; 5 Department of Medical Physics and Medical Bioengeneering, Sahlgrenska University Hospital, 41345 Gothenburg, Sweden; 6 Laboratory of Radiochemistry, Paul Scherrer Institute, 5232 Villigen-PSI, Switzerland; 7 Department of Radiation Oncology, University Hospital of Bern, 3010 Bern, Switzerland; 8 Department of Physics, ETH Zurich, 8093 Zurich, Switzerland

**Keywords:** proton therapy, targeted radionuclide therapy, PET imaging, folate receptor, PSMA, ^177^Lu, combination therapy

## Abstract

Proton therapy (PT) is a treatment with high dose conformality that delivers a highly-focused radiation dose to solid tumors. Targeted radionuclide therapy (TRT), on the other hand, is a systemic radiation therapy, which makes use of intravenously-applied radioconjugates. In this project, it was aimed to perform an initial dose-searching study for the combination of these treatment modalities in a preclinical setting. Therapy studies were performed with xenograft mouse models of folate receptor (FR)-positive KB and prostate-specific membrane antigen (PSMA)-positive PC-3 PIP tumors, respectively. PT and TRT using ^177^Lu-folate and ^177^Lu-PSMA-617, respectively, were applied either as single treatments or in combination. Monitoring of the mice over nine weeks revealed a similar tumor growth delay after PT and TRT, respectively, when equal tumor doses were delivered either by protons or by β¯-particles, respectively. Combining the methodologies to provide half-dose by either therapy approach resulted in equal (PC-3 PIP tumor model) or even slightly better therapy outcomes (KB tumor model). In separate experiments, preclinical positron emission tomography (PET) was performed to investigate tissue activation after proton irradiation of the tumor. The high-precision radiation delivery of PT was confirmed by the resulting PET images that accurately visualized the irradiated tumor tissue. In this study, the combination of PT and TRT resulted in an additive effect or a trend of synergistic effects, depending on the type of tumor xenograft. This study laid the foundation for future research regarding therapy options in the situation of metastasized solid tumors, where surgery or PT alone are not a solution but may profit from combination with systemic radiation therapy.

## 1. Introduction

Cancer in patients often has two disease components: loco-regional and disseminated. The primary tumor and immediate microscopic spread in the surrounding tissue is referred to as loco-regional disease, whereas disseminated disease can typically be metastatic in nature or disease that has spread to remote sites through the lymphatic system. As such, different treatment strategies often need to be applied to these different components. 

Proton therapy (PT) is a curative radiation therapy modality for the treatment of loco-regional disease, which makes use of a proton beam delivering a highly-focused radiation dose to the primary tumor. A major advantage over external beam photon radiotherapy is the possibility of a precise deposition of the proton radiation dose, based on the maximum energy loss and final stopping of protons at a specific depth (“Bragg peak”) [1]. Consequently, this spares the normal tissue in the vicinity of the target volume. On the other hand, targeted radionuclide therapy (TRT) is a systemic treatment approach primarily for disseminated disease that makes use of intravenously-applied radioconjugates, which accumulate selectively in cancer tissue due to specific targeting of tumor cell-associated receptors or antigens. This concept is routinely applied in clinics with, for example, somatostatin receptor-targeted peptides (such as Lutathera™, ^177^Lu-DOTATATE, and somatostatin analog [2]) for the treatment of neuroendocrine tumors, as well as with antibodies to target CD20 (e.g., Zevalin™ and ^90^Y-Ibritumomab tiuxetan [3]) for the treatment of Non-Hodgkin’s lymphoma. More recently, the application of the ^177^Lu-PSMA-617 has shown promising results in metastasized prostate cancer patients [4], which prompted the initiation of a Phase III clinical trial (NCT03511664). TRT can be curative for oligo-metastatic disease and has shown benefit to extend the survival time of patients with end-stage disease and provide them with an improved quality of life. Off-target accumulation of radioconjugates may, however, cause a risk of damage to radiosensitive healthy tissue such as the bone marrow and the kidneys, which present the dose-limiting factors in TRT [5,6].

As such, patients with metastatic disease may profit from a therapy paradigm that combines proton beam irradiation of the local tumor and TRT for the treatment of metastases, including micro-metastases that are not detectable with current imaging modalities. This combination could be of particular interest, as the substantially-reduced dose to normal tissues resulting from proton therapy could be of great advantage when combined with a more systemic, and less localized, treatment such as TRT. Such combination therapies have, however, not been extensively investigated to date. On the other hand, several preclinical and clinical studies have been reported in the literature, in which the combination of external photon beam radiation therapy and targeted TRT was investigated [7,8]. 

The unique situation at the Paul Scherrer Institute, where the Center for Proton Therapy (CPT) and the Center for Radiopharmaceutical Sciences (CRS) are co-localized, offers the possibility to assess these combined treatments. At CRS, a major research focus has been the development and application of folate-based radioligands for targeting folate receptor (FR)-positive tumor types, as well as radioligands for targeting prostate-specific membrane antigen (PSMA)-expressing prostate cancer [9,10,11,12]. FR- and PSMA-targeting agents have been developed at CRS and optimized in terms of their pharmacokinetic properties to make them highly effective for TRT, as demonstrated in preclinical experiments [12,13,14]. Both types of small-molecular mass radioligands have potential for future clinical translation. Similarly, CPT has established itself in the international community as the state-of-the-art proton therapy center, largely due to its pioneering gantry technologies and the development of Pencil Beam Scanning (PBS) [15,16]. The application of PBS to treat cancer patients is world-renowned and has demonstrated its clinical efficacy in a significant number of successfully-treated individuals, as documented in several publications [17,18,19]. One of the most successful programs of CPT is, however, the fixed horizontal beam line OPTIS2, which has been used for the treatment of ocular melanoma and other eye-located tumors since 1984. Using single- and double-scattered-collimated beams, proton treatments at OPTIS2 result in an overall local control of more than 98% after five years [20,21]. 

The aim of this study was to investigate the potential of combining PT and TRT in a preclinical setting, with the aim of establishing equi-equivalent dose levels between the two modalities and to additionally investigate potential additive or synergistic effects. In this proof-of-concept study with tumor-bearing mice, PT and ^177^Lu-based TRT were, therefore, applied separately, as well as in combination, to two different tumor mouse models using ^177^Lu-folate developed at CRS or the clinically-applied ^177^Lu-PSMA-617.

## 2. Materials and Methods 

### 2.1. In Vivo Experiments

In vivo experiments were performed by respecting all applicable international, national, and/or institutional guidelines for the care and use of animals. In particular, all animal experiments were carried out according to the guidelines of Swiss Regulations for Animal Welfare. The preclinical studies were ethically approved by the Cantonal Committee of Animal Experimentation and permitted by the responsible cantonal authorities (No. 75691, July 2016). Athymic female nude mice (CD-1) at the age of 5–6 weeks, obtained from Charles River Laboratories (Sulzfeld, Germany), were used for studies involving ^177^Lu-folate. In this case, mice were fed with a folate-deficient rodent diet (ssniff Spezialdiäten GmbH, Soest, Germany). BALB/c nude mice at the age of 5–6 weeks were also obtained from Charles River Laboratories (Sulzfeld, Germany) and used for studies involving ^177^Lu-PSMA-617. These mice were fed with a standard rodent diet (Kliba Nafag, Granovit AG, Kaiseraugst, Switzerland).

### 2.2. Preparation of Mice

The details of cell culture in order to prepare the cells for inoculation are reported in the Supplementary Material. KB (cervical carcinoma cell line, subclone of HeLa cells, ACC-136) and PC-3 PIP tumor cells (subline of the androgen-independent PC-3 human prostate cancer cell line, kindly provided by Prof. Dr. Martin Pomper, Johns Hopkins University School of Medicine, Baltimore, MD, USA) were used in this study. 

Preparation of mice for the therapy studies: CD1 nude mice were inoculated with KB tumor cells (4.5 × 10^6^ cells in 100 μL PBS) in the subcutis of the left shoulder 4 days before therapy start (Study I). BALB/c nude mice were prepared by inoculation of PC-3 PIP tumors (4.0 × 10^6^ cells in 100 µL Hank’s balanced salt solution; HBSS) in the subcutis of the left shoulder 6 days before therapy start (Study II).

Preparation of mice for PET imaging: Two CD1 nude mice were inoculated with KB tumor cells (4.5 × 10^6^ cells in 100 µL PBS) into the subcutis of each shoulder. Two BALB/c nude mice were inoculated with PC-3 PIP tumor cells (4.0 × 10^6^ in 100 µL HBSS) into the subcutis of each shoulder, respectively. In both mouse models, PET/CT imaging of the tissue, activated by proton irradiation, was performed 16 days after tumor cell inoculation.

### 2.3. Irradiation Station for Mice

**Figure 1:**
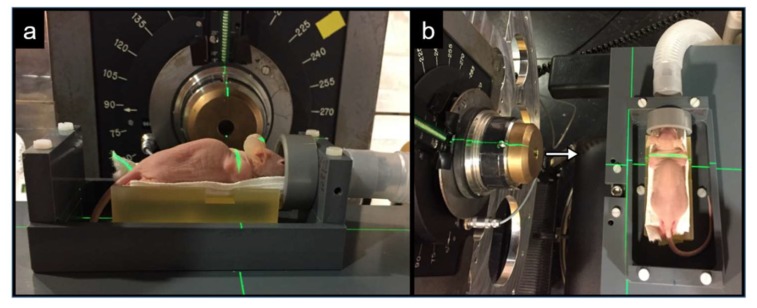
Picture of an anesthetized tumor-bearing mouse positioned on the irradiation platform installed at OPTIS2 at CPT for proton irradiation. (**a**) Frontal view with beam nozzle in the center of the picture and the inhalation anesthesia on the right side. (**b**) Top view with the beam nozzle on the left side and the mouse bed on the right side. Direction of beam is indicated by a white arrow.

A mouse irradiation platform was fabricated and positioned at OPTIS2 of CPT for proton irradiations. A support for the mouse was designed and built to fit the requirements of OPTIS2 geometry. The support was fixed to a metallic device which could be moved with 6 degrees of freedom by the robotic chair used for patient treatment. The beam was shaped according to the dimension of each tumor using cylindrical copper collimators. The compact design of the support allowed the placing of the mouse as close as possible to the collimators, thereby allowing an accurate position with an error margin smaller than 1 mm. Two co-axial tubes placed in front of the mouse head were designed to provide inhalation anesthesia (Figure 1). 

### 2.4. Irradiation of Tumor-Bearing Mice with Protons

The set-up of the irradiation facility for the mice and the precise procedure of the experiments are described in the Supplementary Material. The aim of the experimental set-up was to obtain a homogeneous absorbed dose to the tumor and minimize the absorbed dose to the surrounding tissue. A homogeneous dose was delivered to cover the entire tumor, but sparing the surrounding tissues. All irradiations were performed using a 2.5-mm safety margin to account for possible inaccuracies during positioning, movement of the mouse and other uncertainties, as is used clinically with this facility. The procedure was simplified by considering the tumors in the mice as spheres with a diameter equal to the biggest diameter of the actual tumor, as measured the day before the irradiation. Both mean absorbed dose and range (the deepest point of treatment) of the proton beam were specified for each individual mouse. An extra 2.5-mm posterior margin was applied, to account for the distal uncertainties of the proton beam. A full modulation of the beam was implemented in all cases, meaning that all the tissue, from the surface of the mouse to the point defined by the range, was irradiated. All parameters were used to create irradiation steering files specific to each mouse, that were individually verified using a test phantom, to ensure the delivery of the treatment prescribed in each case.

Mice were anesthetized by inhalation anesthesia using a mixture of isoflurane and oxygen. Mice were positioned for the irradiation by using both in-room mounted lasers and calibrated images acquired by the beam eye view camera installed in the OPTIS2 nozzle. This allowed the adjust of the position of the robotic chair to which the mouse bed was fixed until the center of the tumor appeared in the center of the field. Once the mouse was positioned, the irradiation was initiated by delivering the prescribed dose in 20–40 s.

### 2.5. Preparation and Application of ^177^Lu-Labeled Radioligands 

The chemical structures of the ligands and detailed methods of radiolabeling, including quality control, are reported in the Supplementary Material (Figure S1). The development of the DOTA-folate conjugate (previously referred to as “cm10”) and ^177^Lu-labeling was previously published by Siwowska et al. [22]. Radiolabeling of PSMA-617 was carried out as previously reported by Benešová et al. [11]. The respective biodistribution data of these radioligands in KB and PC-3 PIP tumor mouse models, respectively, are summarized in the Supplementary Material (Table S1 [22] and Table S2 [11]). ^177^Lu-folate and ^177^Lu-PSMA-617, respectively, were prepared at molar activities that resulted in a ligand quantity of 1 nmol per mouse in order to guarantee equal tissue distribution of the radioligands, irrespective of the injected activity.

### 2.6. Dosimetric Calculations for TRT 

In contrast to PT, where the delivered dose distribution can be precisely calculated and measured as part of the standard quality assurance program of the OPTIS2 facility, the dosimetry of TRT is more challenging. As such, biodistribution data, previously published for ^177^Lu-cm10 [22] and ^177^Lu-PSMA-617 [11], respectively, were converted to non-decay-corrected data and used to calculate the mean absorbed doses to the KB and PC-3 PIP tumors, respectively, as previously reported [22]. The mean specific absorbed dose (Gy/MBq) to the tumor xenografts was calculated by multiplication of the time-integrated activity concentration by the emitted electron energy per decay, the absorbed fraction of the emitted electron energy, and a conversion factor. In the case of ^177^Lu-folate, linear integration of the first time points was used for the time-integrated activity concentration between 0 and 24 h post injection (p.i.). The time-integrated activity concentration from 24 h to infinity was calculated by integration of a mono-exponential function. In the case of ^177^Lu-PSMA-617, a linear function was used for the time interval 0–8 h p.i. and a bi-exponential function for the time points 8–196 h p.i. for the tumor. The time-integrated activity concentration was obtained by integration to infinity.

The absorbed fractions for the tumors (40−50 mg) were assessed by Monte Carlo simulations using PENELOPE-2014 [23]. In the simulations, a uniform activity distribution within the tumors was assumed. The emitted electron energy per decay for ^177^Lu was 147 keV (www.nndc.bnl.gov). 

### 2.7. Therapy Studies

Two therapy studies were performed with mice irradiated with protons (PT) or treated with ^177^Lu-folate or ^177^Lu-PSMA-617 (TRT), respectively, or the combination of these two modalities. The dose to the tumors was chosen based on our experience with TRT using ^177^Lu-folate and ^177^Lu-PSMA-617, respectively. The monotherapy with either therapy modality was aimed at delaying the tumor growth, but not to eradicate tumor xenografts entirely in order to enable the determination of potential additive or synergistic effects of the combination therapy. In each experiment, four groups of mice were included and treated at Day 0 of the study. The first group of mice underwent sham irradiation (without delivering proton irradiation) and was injected with only saline (Group A: control). The second and third groups, respectively, were treated with protons only (Group B: PT) or with ^177^Lu-folate or ^177^Lu-PSMA-617 only (Group C: TRT). The fourth group received the combination therapy (Group D: PT and TRT) (Table 1). 

In Study I, PT was applied at doses of 15 Gy and 7.5 Gy for single and combination therapy, respectively, and TRT was applied at 17 MBq ^177^Lu-folate (corresponding to 15 Gy) and 8.5 MBq ^177^Lu-folate (corresponding to 7.5 Gy) for single and combination therapy, respectively. In Study II, PT was applied at doses of 10 Gy and 5 Gy for single and combination therapy, respectively, and TRT was applied at 2.5 MBq ^177^Lu-PSMA-617 (corresponding to 10 Gy) and 1.25 MBq ^177^Lu-folate (corresponding to 5 Gy) for single and combination therapy, respectively. The radioligands were applied intravenously in a lateral tail vein in a volume of 100 mL saline.

The mice were monitored by measuring body weights and the tumor size every other day over 9 weeks. Mice were euthanized when pre-defined endpoint criteria (see below) were reached, or when the study was terminated at Day 63. The relative body weight (RBW) was defined as [BW_x_/BW_0_], where BW_x_ is the body weight in grams at a given Day x and BW_0_ the body weight in grams at Day 0. The tumor dimension was determined by measuring the longest tumor axis (L) and its perpendicular axis (W) with a digital caliper. The tumor volume (V) was calculated according to the equation [V = 0.5 × (L × W^2^)]. The relative tumor volume (RTV) was defined as [TV_x_/TV_0_], where TV_x_ is the tumor volume in mm^3^ at a given Day x and TV_0_ the tumor volume in mm^3^ at Day 0.

The endpoint criteria were set according to the size of the mouse strain. In Study I, they were defined as: (i) a tumor volume >1000 mm^3^; (ii) body weight loss of ≥15%; (iii) tumor volume of ≥900 mm^3^ and body weight loss of ≥10%; or (iv) signs of unease and discomfort. The endpoint criteria in Study II were defined as: (i) a tumor volume ≥800 mm^3^; (ii) body weight loss of ≥15%; (iii) tumor volume of ≥700 mm^3^ and body weight loss of ≥10%; or (iv) signs of unease and discomfort.

### 2.8. Assessment of the Therapy Studies

The efficacy of each treatment modality alone or in combination was expressed as the tumor growth delay (TGD_x_), which was calculated as the time required for the tumor volume to increase x-fold over the initial volume at Day 0. The tumor growth delay index [TGDI_x_ = TGD_x_(T)/TGD_x_(C)] was calculated as the TGD_x_ ratio of treated mice (T) over control mice (C) for a 2-fold (x = 2, TGD_2_) and 5-fold (x = 5, TGD_5_) increase of the initial tumor volume. The median survival was calculated using GraphPad Prism software (version 7). 

The data (average survival time, TGDI_2_ and TGDI_5_) were analyzed for significance as indicated in Section 3 using a one-way ANOVA with Tukey’s multiple comparison post-test using GraphPad Prism software (version 7). A value of *p* < 0.05 was considered statistically significant. Survival of mice was assessed using Kaplan–Meier curves to determine median survival of mice of each group using Graph Pad Prism software (version 7).

### 2.9. PET Imaging

PET/CT scans were performed using a small-animal bench-top PET/CT scanner (G8, Perkin Elmer, Waltham, MA, USA.) in order to qualitatively estimate the delivered dose distribution by PT [24]). Due to the compact size of this scanner, it was possible to transport it from CRS to CPT to the OPTIS2 treatment room. PET/CT imaging studies were performed with mice bearing KB tumors or PC-3 PIP tumors, respectively, on each shoulder. The left tumor xenograft of each mouse was irradiated with protons at a dose of 20 Gy. Immediately afterwards, the mouse was placed on the PET animal bed and moved into the scanner (Figure S2). Static whole-body PET scans of 10 min duration were performed followed by a CT of 1.5 min. During the in vivo scans, the mice were anesthetized with a mixture of isoflurane and oxygen. Reconstruction of acquired data was performed using the software of the provider of the G8 scanner. All images were prepared using VivoQuant post-processing software (version 3.0, inviCRO Imaging Services and Software, Boston, MA, USA). A Gauss post-reconstruction filter was applied (FWHM = 1 mm) to the images, which were presented with the scale adjusted by cutting 5% of the lower scale in order to allow visualization of the most important organs and tissues.

## 3. Results

### 3.1. Dosimetry Estimation for TRT Using ^177^Lu-Folate and ^177^Lu-PSMA-617

The mean absorbed tumor dose for ^177^Lu-cm10 was 0.89 Gy/MBq, as previously published by Siwowska et al. [22], which corresponded to an absolute dose of ~7.5 Gy and ~15 Gy when injecting 8.5 MBq or 17 MBq ^177^Lu-folate, respectively. The mean absorbed tumor dose for ^177^Lu-PSMA-617 was 4.0 Gy/MBq, which corresponded to an absolute dose of ~5 Gy and ~10 Gy when injecting 1.25 MBq or 2.5 MBq ^177^Lu-PSMA-617, respectively. The numbers calculated for the mean absorbed tumor dose delivered by TRT can be considered as a dose estimation rather than an exact value, as is the case when using external proton beam radiation therapy (PT).

### 3.2. Therapy Studies

#### 3.2.1. Therapy Study I: Combination Proton Therapy and ^177^Lu-Folate Therapy

**Figure 2:**
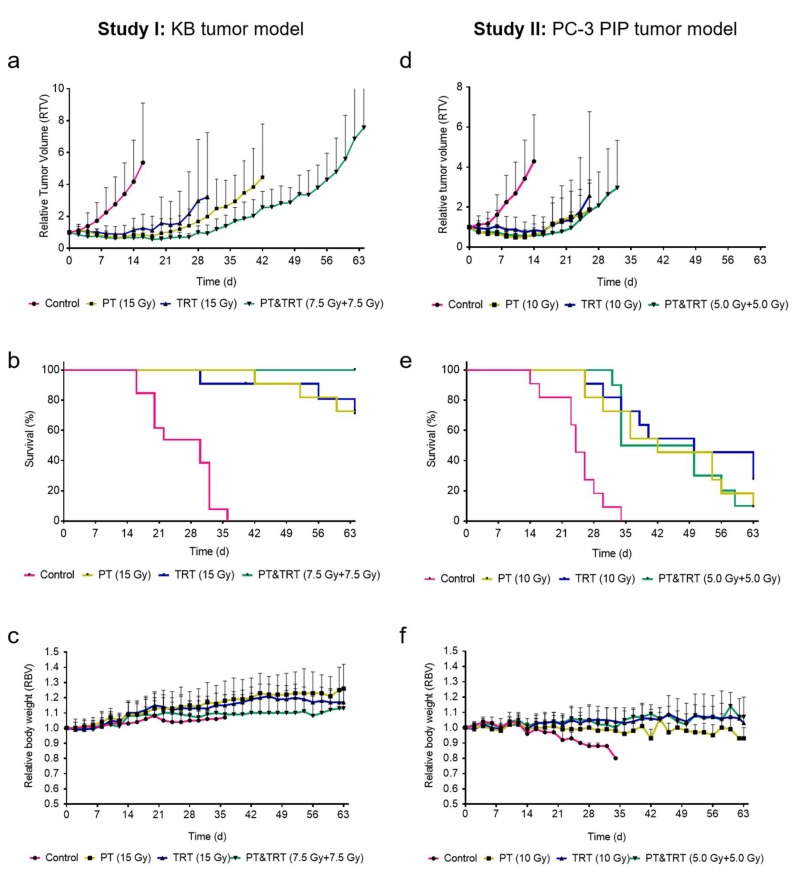
Graphs showing average relative tumor volume of each group until the first mouse of each group had to be euthanized, the median survival and the relative body weight of mice of: Study I (**a**–**c**); and Study II (**d**–**f**). Control mice (control; pink), mice treated with protons (PT; yellow), mice treated with ^177^Lu-folate (TRT; blue) and the combination of these modalities (PT and TRT; green). (**a**,**d**) Tumor growth curves shown as average of all mice of one group until the first mouse of each group had to be euthanized; (**b**,**e**) Kaplan–Meier survival curves; and (**c**,**f**) relative body weights.

In the first study, CD1 nude mice inoculated with KB tumor cells, were treated with PT, TRT or a combination of both. A relatively fast KB tumor growth was observed in untreated control mice (sham-irradiated and injected with saline; Group A). This resulted in two mice to reach the endpoint at Day 16, while the last mouse was euthanized at Day 36. The tumor growth of treated mice was delayed and resulted in the first mouse of Group B to reach an endpoint at Day 42 while in Group C this was the case at Day 30. In Groups B and C, 8 mice out of 11 were still alive at study end on Day 64. Interestingly, none of the mice of the combination therapy (Group D) reached an endpoint within the 64 days of monitoring (Table 2 and Figure 2a). As compared to control mice of Group A, the time to reach a relative tumor volume of 2 (TGDI_2_) was, with 3.5-, 3.2- and 4.2-fold, significantly (*p* < 0.05) longer for mice of the treated groups (Groups B–D). A significant difference among the TGDI_2_ values among groups of treated mice was only observed for Groups C and D (*p* < 0.05). The same significance was also found for the calculated TGDI_5_. The median survival time of control mice (Group A) was 30 days, however, in all other groups (Groups B–D), the median survival remained undefined since more than 50% of the mice were still alive at study end (Table 2 and Figure 2b). The body weight of mice increased in all groups over time, indicating that the therapies were well tolerated (Figure 2c). 

#### 3.2.2. Therapy Study II: Combination of Proton Therapy and ^177^Lu-PSMA-617 Therapy

In the second study, BALB/c nude mice inoculated with PC-3 PIP-cells were treated with PT, TRT or a combination of both. A relatively fast tumor growth was observed in untreated control mice (sham-irradiated and injected with saline; Group A), which led the first mouse to reach the endpoint already on Day 14. The tumor growth of all treated groups was slower, resulting in the first mice to be euthanized at Day 26 (Groups B and C) and Day 32 (Group D), respectively. While in Group A all mice reached an endpoint within the 36 days of investigation, one mouse in each of Groups B and D and three mice in Group C were still alive at the end of the study (Table 2 and Figure 2d). To reach a relative tumor volume of 2 (TGDI_2_), the time was 3.3–3.8-fold (*p* < 0.05) longer for treated mice (Groups B–D) as compared to control mice of Group A. There was, however, no significant difference (*p >* 0.05) determined among each of the treated groups (Groups B–D; Table 2). The same findings held true for the TGDI_5_ which was significantly (*p* < 0.05) increased in treated groups (Groups B–D) as compared to control mice of Group A, but no significant difference (*p* > 0.05) was found among the treated groups (Groups B–D). The median survival time of mice treated with 10 Gy of either therapy modality (Groups B and C) or the combination (Group D) was in the range of 42–50 days which was clearly longer than for control mice (Group A: median survival, 24 days). The longest median survival time was observed in mice that received ^177^Lu-PSMA-617 (Group C: 50 days) (Table 2 and Figure 2e). In line with these findings, the average survival was significantly longer (*p* < 0.05) for treated mice than for control mice, but there was no significant difference in the average survival among the single therapy groups (Groups B–D). The body weight was relatively constant in treated mice, but control mice lost body weight over time, presumably due to the increasing tumor burden (Figure 2f).

### 3.3. PET-Based Imaging of Tissue Activation after Proton Irradiation

**Figure 3:**
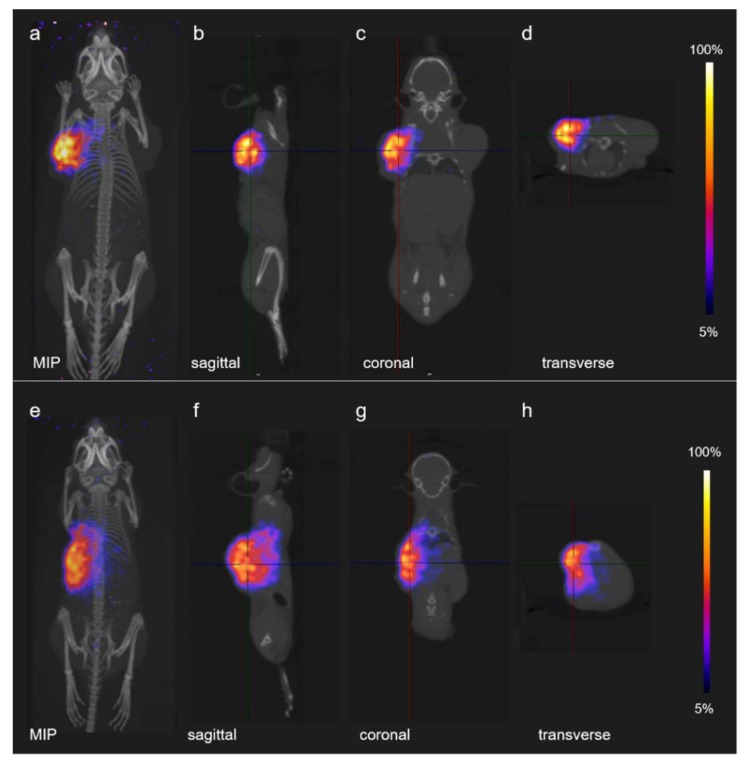
PET/CT images of proton induced activation in mice: (**a**–**d**) PET/CT images of a mouse bearing KB tumor xenografts; and (**e**,**f**) PET/CT images of a mouse bearing PC-3 PIP tumor xenografts. For both cases, the same proton dose of 20 Gy was delivered to the implanted tumor located on the left side of the mouse: (**a**,**e**) maximum intensity projections (MIP); (**b**,**f**) sagittal sections; (**c**,**g**) coronal sections; and (**d**,**h**) transverse sections.

Tissue activation upon irradiation of the mice with protons was demonstrated using PET/CT. Mice were kept still under anesthesia and scanned immediately (approximately 3 min) after proton irradiation. PET/CT images with either tumor mouse model showed highly focused regions of tissue activation in the tumor located on the left side of the animal, qualitatively demonstrating the high dose conformation to the tumor of the proton treatments. On the other hand, no PET signal was detected in the tumor which was not irradiated, located on the right shoulder of the mouse (Figure 3).

## 4. Discussion

In this study, the combination of PT with TRT was explored using two different xenograft mouse models. The therapy experiments showed comparable results of PT and TRT, applied at equal dose or combined while using half of the dose. Interestingly, the results showed a trend of beneficial effect when combining PT and TRT in the KB tumor model. This was demonstrated by significantly increased TGDI_2_ and TGDI_5_ for mice receiving the combination therapy, as compared to the mice treated with either PT or TRT, respectively. At this stage, there is no plausible explanation for these findings. It was also not confirmed for the PC-3 PIP tumor-bearing mice, where an additive effect rather than a synergistic effect was observed in the group of mice which received the combination therapy. Interestingly, the dose calculation for the TRT was quite accurate even though there was a number of uncertainties regarding the values on which the calculation was based. This concerns the exact quantity of injected activity, inter-individually variable distribution profiles of the radioligand and potentially inhomogeneously distributed activity in the tumor tissue. The calculation of the absorbed dose to tumors after radionuclide therapy should, thus, be regarded as an approximation rather than an exact dose calculation. This is in clear contrast to PT, where the absorbed dose can be exactly determined. 

The phenomenon of tissue activation by proton irradiation and, consequently, the production of short-lived PET radionuclides was previously investigated and the production of short-lived PET radionuclides demonstrated [25]. It was found that the emitted positrons predominantly stemmed from ^15^O, while ^11^C contributed to a smaller extent for the first ~9 min after irradiation. At later time points (>10 min after proton irradiation), ^11^C appeared to be the predominant source of positron emission, while the production of ^13^N played a minor role in this regard [25]. The major nuclear reaction channels for proton-induced positron emitter production were previously reported in the literature (Table S3) [25]. The production of ^15^O occurs via the ^16^O(p,pn)^15^O nuclear reaction, whereas the production of ^11^C can occur by different nuclear reactions depending on the proton energy [25]. In the study presented herein, visualization of activated tissue was successfully achieved immediately after proton irradiation of the tumors. The results demonstrate the feasibility of precisely irradiating small tumor xenografts with protons, without affecting neighboring healthy tissue.

Given that PT is a modality for loco-regional treatments, whereas TRT is for systemic treatment of oligo-metastatic cancer patients, their combination may be both beneficial as well as complimentary. As an example, lung cancer, as the leading cancer killer worldwide, could profit from such combination therapy. In contrast to external photon beam therapy, PT of lung cancer patients has shown relatively low rates of toxicity, however, the survival benefits have been unclear [26]. This may be due to the existence of untreated distant metastases. The metastatic disease could be addressed by TRT using therapeutic radionuclides (e.g., ^177^Lu, ^225^Ac) and a ligand that targets an appropriate tumor-associated cell surface marker, such as the FR or the somatostatin receptor.

In this study, the foundation has been laid for future preclinical studies with mice to investigate the concept of combining PT and TRT. In this regard, it would be of interest to investigate mouse models not only with a solid tumor, but also with metastases to evaluate whether combining localized therapy (PT) and systemic therapy (TRT) would complement each other in a clinically-relevant patient situation.

Other than the question with regard to the size of tumor lesions and accessibility for the relevant radiation dose to be delivered, the topic of radiobiological and immunological effects as a response to different radiation types has not yet been extensively investigated. It may be of high importance, however, in view of a future clinical translation. Understanding the dose response of tumor cells and the tumor microenvironment and normal tissue, respectively, has received renewed interest, particularly in view of combination therapies with immunological approaches [27]. The same holds true for TRT, where different types of particle radiation (α, β^−^, and Auger electrons) are being studied with regard to their radiobiological effects in tumor tissue [28]. In future, it is planned to explore and compare the therapy concepts in this regard using syngeneic and spontaneous tumor mouse models, with an intact immune system, enabling the investigation of immunological responses to different radiation modalities.

## 5. Conclusions

In this preclinical pilot study, additive, or even a trend of synergistic effects depending on the tumor xenograft type, have been demonstrated by combining PT and TRT. The current study has laid essential foundations for future research regarding combination therapies of different radiation therapies, as well as regarding a better understanding of radiobiological effects of these and further radiation types. The research, which we intend to pursue in future, may be fundamental to identifying the most effective application scheme of these combinations as an essential precondition in view of a clinical translation.

## Figures and Tables

**Table 1:** Study designs of the therapies using PT and TRT through use of ^177^Lu-folate (Study I) or ^177^Lu-PSMA-617 (Study II).

**Study I: KB Tumor Mouse Model**				
**Groups**	**A: Control**	**B: PT**	**C: TRT**	**D: PT and TRT**
Proton Irradiation	Sham irradiation	Irradiation: 15 Gy	Sham irradiation	Irradiation: 7.5 Gy
^177^Lu-Folate Treatment	Saline injection	Saline injection	^177^Lu-folate 15 Gy (17 MBq) 1 nmol/mouse	^177^Lu-folate 7.5 Gy (8.5 MBq) 1 nmol/mouse
Number of Mice	*n* = 13	*n* = 11	*n* = 11	*n* = 11
**Study II: PC-3 PIP Tumor Mouse Model**				
**Groups**	**A: Control**	**B: PT**	**C: TRT**	**D: PT and TRT**
Proton Irradiation	Sham irradiation	Irradiation: 10 Gy	Sham irradiation	Irradiation: 5.0 Gy
^177^Lu-PSMA-617 Treatment	Saline injection	Saline injection	^177^Lu-folate: 10 Gy (2.5 MBq) 1 nmol/mouse	^177^Lu-folate: 5.0 Gy (1.25 MBq) 1 nmol/mouse
Number of Mice	*n* = 11	*n* = 11	*n* = 11	*n* = 11

**Table 2:** Parameters characterizing the efficacy of the applied therapy. (A) Study I: Investigation of KB tumor-bearing CD1 nude mice. (B) Study II: Investigation of PC-3 PIP tumor-bearing BALB/c nude mice.

**Study I: KB Tumor Mouse Model**							
**Group**	**Tumor Dose (Gy)**	**First Mouse Euthanized (Day)**	**Last Mouse Euthanized (Day)**	**Median Survival (days)**	**Average Survival (days)**	**TGDI_2_**	**TGDI_5_**
A: Saline	-	16	36	30	27 ± 9 (*n* = 13)	1.0 ± 0.4 (*n* = 13)	1.0 ± 0.3 (*n* = 13)
B: PT	15	42	64 (study end)	not reached	*n* = 3 (endpoint) ^1^ *n* = 8 (alive) ^2^	3.5 ± 1.1 (*n* = 11)	2.7 ± 0.7 (*n* = 9)
C: TRT	~15	30	64 (study end)	not reached	*n* = 3 (endpoint) ^1^ *n* = 8 (alive) ^2^	3.2 ± 1.1 (*n* = 10)	2.4 ± 0.9 (*n* = 10)
D: PT & TRT	7.5 + ~7.5	none	64 (study end)	not reached	*n* = 0 (endpoint) ^1^ *n* = 11 (alive) ^2^	4.2 ± 0.6 (*n* = 11)	3.2 ± 0.2 (*n* = 11)
**Study II: PC-3 PIP Tumor Mouse Model**							
**Group**	**Tumor Dose (Gy)**	**First Mouse Euthanized (Day)**	**Last Mouse Euthanized (Day)**	**Median Survival (days)**	**Average Survival (days)**	**TGDI_2_**	**TGDI_5_**
A: Saline	-	14	34	24	26 ± 6 (*n* = 11)	1.0 ± 0.5 (*n* = 11)	1.0 ± 0.4 (*n* = 10)
B: PT	10	26	64 (study end)	42	*n* = 10 (endpoint) ^1^ *n* = 1 (alive) ^2^	3.3 ± 1.5 (*n* = 11)	2.3 ± 0.4 (*n* = 8)
C: TRT	~10	26	64 (study end)	50	*n* = 8 (endpoint) ^1^ *n* = 3 (alive) ^2^	3.8 ± 2.1 (*n* = 9)	1.7 ± 0.6 (*n* = 6)
D: PT & TRT	5.0 + ~5.0	32	64 (study end)	42	*n* = 10 (endpoint) ^1^ *n* = 1 (alive)	3.6 ± 1.3 (*n* = 11)	2.1 ± 0.6 (*n* = 11)

^1^ Mice that have reached an endpoint; ^2^ Mice that were still alive at the end of the study.

## Supplementary Materials

The following are available online at https://www.mdpi.com/1999-4923/11/9/450/s1: Methods of cell culture, set-up of irradiation station, methods for preparation of the radioligands for TRT and PET/CT imaging; Figure S1: Chemical structures of the tumor targeting agents (folate and PSMA-617); Figure S2: Sketch of the procedure of PET/CT imaging immediately after proton irradiation. Tables S1 and S2: previously-published biodistribution data of the radioligands; Table S3. Major nuclear reaction channels for proton induced positron emitter productions.
