# Supplementary material for: Combination of Proton Therapy and Radionuclide Therapy in Mice: Preclinical Pilot Study at the Paul Scherrer Institute
Source: Pharmaceutics. 2019 Sep 2;11(9):450. doi: 10.3390/pharmaceutics11090450 (PMC6781294; doi:10.3390/pharmaceutics11090450)
Supplement: Supplementary file 1 [file pharmaceutics-11-00450-s001.pdf]

pharmaceutics

Supplementary Materials: Combination of Proton Therapy and Radionuclide Therapy in Mice: Preclinical Pilot Study at the Paul Scherrer Institute

Cristina Müller, Maria De Prado Leal, Marco D. Dominietto, Christoph A. Umbricht, Sairos Safai, Rosalind L. Perrin, Martina Egloff, Peter Bernhardt, Nicholas P. van der Meulen, Damien C. Weber, Roger Schibli and Antony J. Lomax

1. Cell Culture

KB cells (cervical carcinoma cell line, subclone of HeLa cells, ACC-136) were purchased from the German Collection of Microorganisms and Cell Cultures (DSMZ, Germany). Cells were cultured in folate-deficient RPMI medium (FFRPMI, Cell Culture Technologies GmbH, Gravesano, Switzerland) supplemented with 10% fetal calf serum, L-glutamine and antibiotics. Routine cell culture was performed twice a week using trypsin for detachment of the cells.

Sublines of the androgen-independent PC-3 human prostate cancer cell line, PSMA-positive PC-3 PIP and PSMA-negative PC-3 flu cells, were kindly provided by Prof. Dr. Martin Pomper (Johns Hopkins University, Baltimore MD, USA). The cells were cultured in RPMI-1640 medium supplemented with 10% fetal calf serum, L-glutamine and antibiotics. Puromycin (2 μg/mL) was added to the culture medium in order to maintain PSMA expression as previously reported [1].

2. Set-up of an animal bed and irradiation of tumor-bearing mice at OPTIS2 at CPT

A custom-made animal bed was designed and constructed in-house for an exact placement of the mouse in the beam path. The support was fixed onto a metallic frame and secured on the robotic chair of OPTIS2, allowing for sub-millimeter accuracy in the positioning of the subjects. The animal bed was connected with tubing for inhalation anesthesia using standard device and isoflurane vaporizer. A warming system of the bed was not necessary, since the total time necessary to adjust the beam including irradiation time (~20–40 s) was less than 10 min.

Mice were irradiated with a fixed horizontal proton beam at OPTIS2 facility at CPT. The aim of the experimental set-up was to obtain a homogenous absorbed dose to the tumor and minimize the absorbed dose to the surrounding tissue. In clinical practice, the scattered proton beam needs to be collimated by using patient-specific copper collimators. This way, the beam is shaped conformally around the projection of the tumor outline in the XY-plane (perpendicular to the direction of the protons), with 2.5 mm of extra margin around said outline to account for possible inaccuracies during the patient positioning, movement of the target and other uncertainties. The experiment was performed by adopting the clinical policy of a 2.5 mm-extra margin to account for possible inaccuracies during positioning, movement of the mouse and other uncertainties. Other than that, the procedure was simplified by considering the tumors of the mice as spheres with a diameter equal to the biggest diameter of the actual tumor, as measured the evening before irradiation. Both dose and range (the deepest point of treatment) of the proton beam were specified for each individual mouse. An extra 2.5 mm posterior margin was applied, to account for the distal uncertainties of the proton beam. Since the inoculated tumor cells grew to superficial tumor xenografts, a full modulation of the beam was implemented in all cases, meaning that all the tissue, from the surface of the mouse to the point defined by the range, was irradiated. All these parameters were used to create irradiation steering files specific to each mouse, that were individually verified using a test phantom, to ensure the delivery of the treatment prescribed in each case.

Mice were anesthetized in an anesthesia box before transfer of each individual mouse to the animal bed, where the mouse nose was placed in the tubing to receive inhalation anesthesia using a

mixture of isoflurane and oxygen. This procedure was done as a simulation (sham irradiation for mice of the control group) or for therapy using proton irradiation (treatment of mice for therapy groups). The positioning of the mice for the irradiation was done by using both in-room mounted lasers and calibrated images acquired by the beam-eye-view camera installed in the OPTIS2 nozzle, adjusting the position of the robotic chair until the center of the tumor appeared in the center of the field. Once the mouse was positioned, the irradiation was initiated by delivering the prescribed dose in 20–40 s.

3. Radioligands used for Targeted Radionuclide Therapy

Preparation of ^{177}Lu-folate: The folate ligand (cm10; hereafter referred to as "folate") used in this study was composed of a folic acid molecule for targeting the folate receptor (FR), a 1,4,7,10-tetraazacyclododecane-1,4,7,10-tetraacetic acid (DOTA) chelator for coordination of the ^{177}Lu and an albumin-binding entity to enhance the blood circulation time and, thereby, improve the tissue distribution profile of the radiofolate (Figure S1a). This ligand was previously developed at CRS [2]. Radiolabeling of the albumin-binding folate (cm10) was performed as previously reported [2] using molar activities as described in the main manuscript (Table 1). No-carrier-added $^{177}LuCl_3$ (0.04 M HCl, obtained from Isotope Technologies Garching ITG GmbH) diluted in 0.05 M HCl was mixed with Na-acetate (0.5 M, pH 8) at the ratio of 5:1 to obtain a solution of pH ~4.5. After the addition of the folate conjugates (stock solutions of 1 mM) the reaction vial was incubated for 10 min at 95 °C. Quality control was performed by reversed-phase high performance liquid chromatography (HPLC; Merck LaChrom HPLC) using a C-18 column (XTerra™ MS, 5µm, 4.6 mm x 150 mm, Waters). The eluent consisted of acetonitrile (solvent A) and 0.1% trifluoroacetic acid in MilliQ water (solvent B). HPLC was run with a gradient of 5–80% A and 95–20% B and used over 15 min with a flow rate of 1.0 mL/min.

Preparation of ^{177}Lu-PSMA-617: PSMA-617 [3], a PSMA-targeting ligand with a DOTA chelator for the coordination of ^{177}Lu was purchased from ABX GmbH, Radeberg, Germany (Figure S1b). Radiolabeling of PSMA-617 was carried out, as previously reported [4] using molar activities as described in the main manuscript (Table 1). The procedure of labeling including quality control was the same as described above for the folate conjugate.

Injection of the radioconjugates for TRT: ^{177}Lu-folate and ^{177}Lu-PSMA-617, respectively, were prepared at specific activities that resulted a ligand quantity of 1 nmol per mouse in order to guarantee equal tissue distribution profile irrespective of the injected activity.

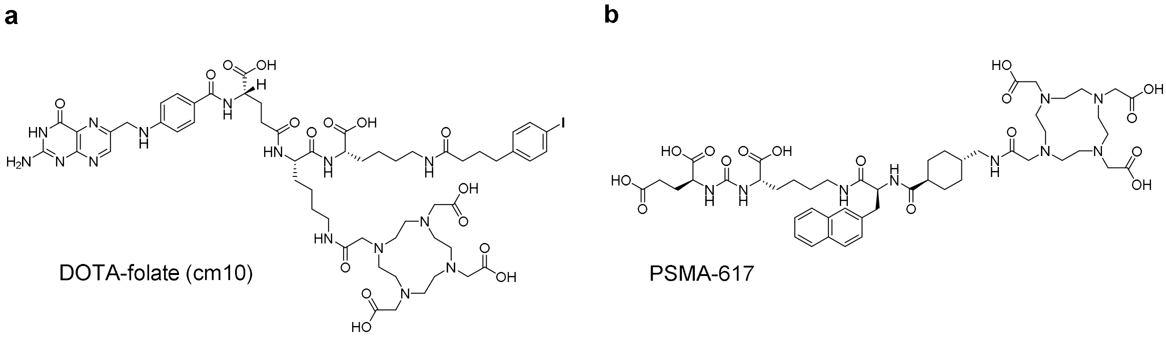

Figure S1. Chemical structures of the tumor targeting agents. **(a)** Chemical structure of the folate conjugate referred to as "cm10". It consists of a folic acid molecule (for targeting the FR), which is conjugated to a DOTA chelator (for coordination of ^{177}Lu) and an albumin-binding entity (enabling binding to albumin and, hence, extend the blood circulation time); **(b)** Chemical structure of PSMA-617 which consists of a glutamate-urea-based PSMA-binding entity and a DOTA chelator connected via a linker entity.

4. Previously-Published Biodistribution Data of ^{177}Lu-Folate and ^{177}Lu-PSMA-617

The data presented in Tables S1 and S2 were previously obtained in our group and published by Siwowska et al. [2] and by Benešová et al. [4], respectively.

Table S1. Decay-corrected biodistribution data of ^{177}Lu-folate (^{177}Lu-cm10) obtained in KB tumor-bearing. The values are presented as percentage of injected activity per gram tissue [% IA/g].

	**^{177}Lu-folate (^{177}Lu-cm10)[2]**			
	**1 h p.i.**	**4 h p.i.**	**24 h p.i.**	**48 h p.i.**
Blood	7.89 ± 1.36	2.34 ± 0.50	0.49 ± 0.08	0.19 ± 0.00
Lung	4.20 ± 0.75	1.57 ± 0.20	0.82 ± 0.16	0.49 ± 0.19
Spleen	1.61 ± 0.19	0.67 ± 0.06	0.55 ± 0.02	0.33 ± 0.05
Kidneys	22.6 ± 3.9	21.9 ± 2.8	28.1 ± 2.7	18.6 ± 0.6
Stomach	1.85 ± 0.42	0.93 ± 0.26	0.59 ± 0.07	0.29 ± 0.06
Intestines	1.37 ± 0.19	0.63 ± 0.07	0.20 ± 0.10	0.23 ± 0.20
Liver	5.00 ± 1.26	3.14 ± 0.36	3.10 ± 0.98	1.86 ± 0.37
Salivary glands	9.79 ± 2.42	5.64 ± 0.26	4.60 ± 1.21	2.43 ± 0.39
Muscle	1.27 ± 0.11	1.12 ± 0.21	1.00 ± 0.21	0.58 ± 0.26
Bone	1.62 ± 0.13	1.27 ± 0.22	0.74 ± 0.31	0.50 ± 0.09
KB tumor	11.1 ± 0.5	14.3 ± 1.9	17.6 ± 2.6	10.1 ± 1.2

The values represent the mean ± S.D. of data from three animals (n = 3).

Table S2. Decay-corrected biodistribution data of ^{177}Lu-folate (^{177}Lu-cm10) obtained in KB tumor-bearing. The values are presented as percentage of injected activity per gram tissue [% IA/g].

	**^{177}Lu-PSMA-617[4]**			
	**1 h p.i.**	**4 h p.i.**	**24 h p.i.**	**48 h p.i.**
Blood	0.50 ± 0.06	0.02 ± 0.33	0.01 ± 0.09	0.01 ± 0.00
Lung	0.57 ± 0.07	0.07 ± 0.08	0.03 ± 0.09	0.02 ± 0.00
Spleen	0.63 ± 0.27	0.15 ± 0.31	0.05 ± 0.11	0.04 ± 0.00
Kidneys	9.75 ± 1.37	3.68 ± 3.20	0.76 ± 0.92	0.35 ± 0.05
Stomach	0.18 ± 0.02	0.08 ± 0.09	0.03 ± 0.03	0.02 ± 0.00
Intestines	0.24 ± 0.00	0.07 ± 0.01	0.04 ± 0.04	0.01 ± 0.00
Liver	0.20 ± 0.04	0.09 ± 0.18	0.07 ± 0.09	0.04 ± 0.01
Salivary glands	0.42 ± 0.30	0.04 ± 0.01	0.02 ± 0.00	0.01 ± 0.01
Muscle	0.25 ± 0.20	0.02 ± 0.06	0.01 ± 0.04	0.01 ± 0.00
Bone	0.21 ± 0.04	0.06 ± 0.07	0.03 ± 0.02	0.03 ± 0.00
PC-3 PIP tumor	44.2 ± 12.0	56.0 ± 8.0	37.3 ± 5.8	28.3 ± 3.8

The values represent the mean ± S.D. of data from three to six animals (n = 3–6).

5. PET Imaging

PET/CT scans were performed using a small-animal bench-top PET/CT scanner (G8, Perkin Elmer, Massachusetts, U.S.A. [5]). Due to the compact size of this scanner, it was possible to transport it from CRS to CPT in order to place it in closest proximity to the proton irradiation station at CPT. PET/CT imaging studies were performed with two mice bearing a KB tumor on each shoulder. The left KB tumor xenograft of each mouse was irradiated with protons to deliver a dose of 20 Gy. Immediately afterwards, the mouse was placed on the PET animal bed and moved into the scanner (Figure S1). Static whole-body PET scans of 10 min duration were performed, followed by a CT of 1.5 min. During the in vivo scans, the mice were anesthetized with a mixture of isoflurane and oxygen. Reconstruction of acquired data was performed using the software of the provider of the G8 scanner. All images were prepared using VivoQuant post-processing software (version 3.0, inviCRO Imaging Services and Software, Boston U.S.A). A Gauss post-reconstruction filter was applied (FWHM = 1 mm) to the images, which were presented with the scale adjusted (by cutting 5% of the lower scale) to allow visualization of the most important organs and tissues.

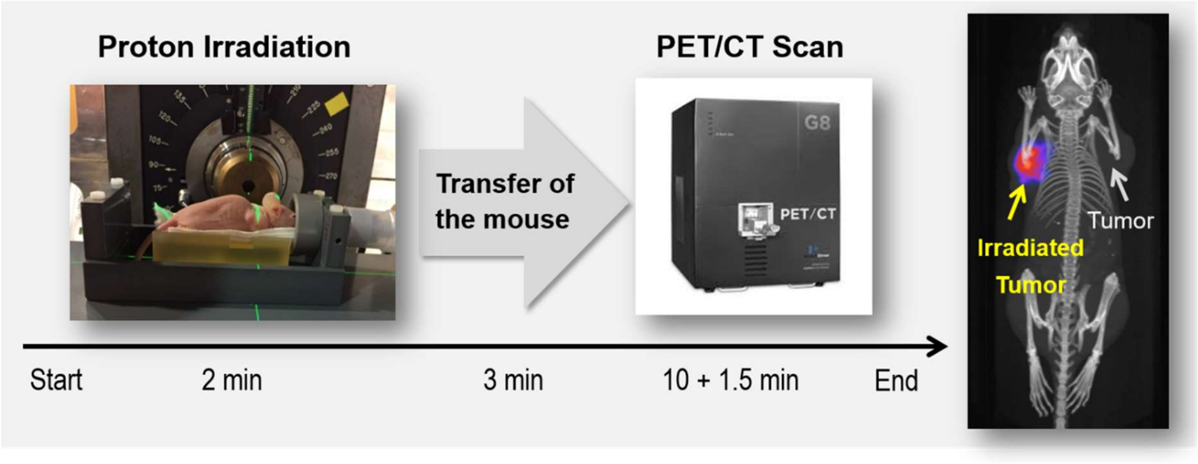

Figure S2. Sketch of the procedure of PET/CT imaging immediately after proton irradiation of the left tumor of the mouse to visualize tissue activation in the irradiated tumor but not in the non-irradiated tumor on the right side.

6. Tissue Activation after Proton Beam Irradiation

According to a study, previously published by Zhu et al., the relevant nuclear reactions that occur in tissue are based on the interaction of protons with ^{16}O, ^{12}C and ^{14}N for as well as ^{31}P and ^{40}Ca in the bones (Table S3).

Table 3. Major nuclear reaction channels for proton induced positron emitter productions.

Radionuclide	Half-life (min)	Nuclear reaction channels	Treshold energy (MeV)
^{15}O	2.04	$^{16}O(p,pn)^{15}O$	16.79
^{11}C	20.4	$^{12}C(p,pn)^{11}C$	20.61
		$^{14}N(p,2p2n)^{11}C$	3.22
		$^{16}O(p,3p3n)^{11}C$	59.64
^{13}N	9.97	$^{16}O(p,2p2n)^{13}N$	5.66
		$^{14}N(p,pn)^{13}N$	11.44
^{30}P	2.50	$^{31}P(p,pn)^{30}P$	19.7
^{38}K	7.64	$^{40}Ca(p,2p2n)^{38}K$	21.2

* Data reproduced from Zhu et al. 2013 Theranostics 10:731-740.

References

1. Umbricht, C.A.; Benešová, M.; Schmid, R.M.; Türler, A.; Schibli, R.; van der Meulen, N.P.; Müller, C. ^{44}Sc-PSMA-617 for radiotheragnostics in tandem with ^{177}Lu-PSMA-617-preclinical investigations in comparison with ^{68}Ga-PSMA-11 and ^{68}Ga-PSMA-617. *EJNMMI Res* **2017**, *7*, 9.
2. Siwowska, K.; Haller, S.; Bortoli, F.; Benešová, M.; Groehn, V.; Bernhardt, P.; Schibli, R.; Müller, C. Preclinical comparison of albumin-binding radiofolates: Impact of linker entities on the in vitro and in vivo properties. *Mol Pharm* **2017**, *14*, 523–532.
3. Benesova, M.; Schäfer, M.; Bauder-Wüst, U.; Afshar-Oromieh, A.; Kratochwil, C.; Mier, W.; Haberkorn, U.; Kopka, K.; Eder, M. Preclinical evaluation of a tailor-made DOTA-conjugated PSMA inhibitor with optimized linker moiety for imaging and endoradiotherapy of prostate cancer. *J Nucl Med* **2015**, *56*, 914–920.
4. Benešová, M.; Umbricht, C.A.; Schibli, R.; Müller, C. Albumin-binding PSMA ligands: Optimization of the tissue distribution profile. *Mol Pharm* **2018**, *15*, 934–946.
5. Gu, Z.; Taschereau, R.; Vu, N.T.; Prout, D.L.; Silverman, R.W.; Lee, J.T.; Chatziioannou, A.F. Performance evaluation of G8, a high-sensitivity benchtop preclinical PET/CT tomograph. *J Nucl Med* **2019**, *60*, 142–149.
